# Supplementary material for: Store-Operated Calcium Entry in Breast Cancer Cells Is Insensitive to Orai1 and STIM1 N-Linked Glycosylation
Source: Cancers (Basel). 2022 Dec 29;15(1):203. doi: 10.3390/cancers15010203 (PMC9818078; doi:10.3390/cancers15010203)
Supplement: Supplementary file 1 [file cancers-15-00203-s001.zip › cancers-2019792-supplementary.pdf]

Supplementary material

Store-Operated Calcium Entry in Breast Cancer Cells Is Insensitive to Orai1 and STIM1 N-linked Glycosylation

Jose Sanchez-Collado [1,†], Joel Nieto-Felipe [1,†], Isaac Jardin [1], Rajesh Bhardwaj [2,‡], Alejandro Berna-Erro [1], Gines M. Salido [1], Tarik Smani [3], Matthias A Hediger [2], Jose J. Lopez [1,*] and Juan A. Rosado [1,*]

[1] Department of Physiology, Institute of Molecular Pathology Biomarkers, University of Extremadura, 10003 Caceres, Spain
[2] Membrane Transport Discovery Lab, Department of Nephrology and Hypertension and Department of Biomedical Research, University of Bern, CH-3010 Bern, Switzerland
[3] Department of Medical Physiology and Biophysic, Institute of Biomedicine of Sevilla, 41013 Sevilla, Spain
* Correspondence: Correspondence: jjlopez@unex.es (J.J.L.); jarosado@unex.es (J.A.R.)
† These authors contributed equally to this work.
‡ Current address: Signal Transduction Laboratory, National Institute of Environmental Health Sciences, NIH, 111 TW Alexander Drive, NC 27709, USA.

This PDF file includes:

Figure S1 y Table 1

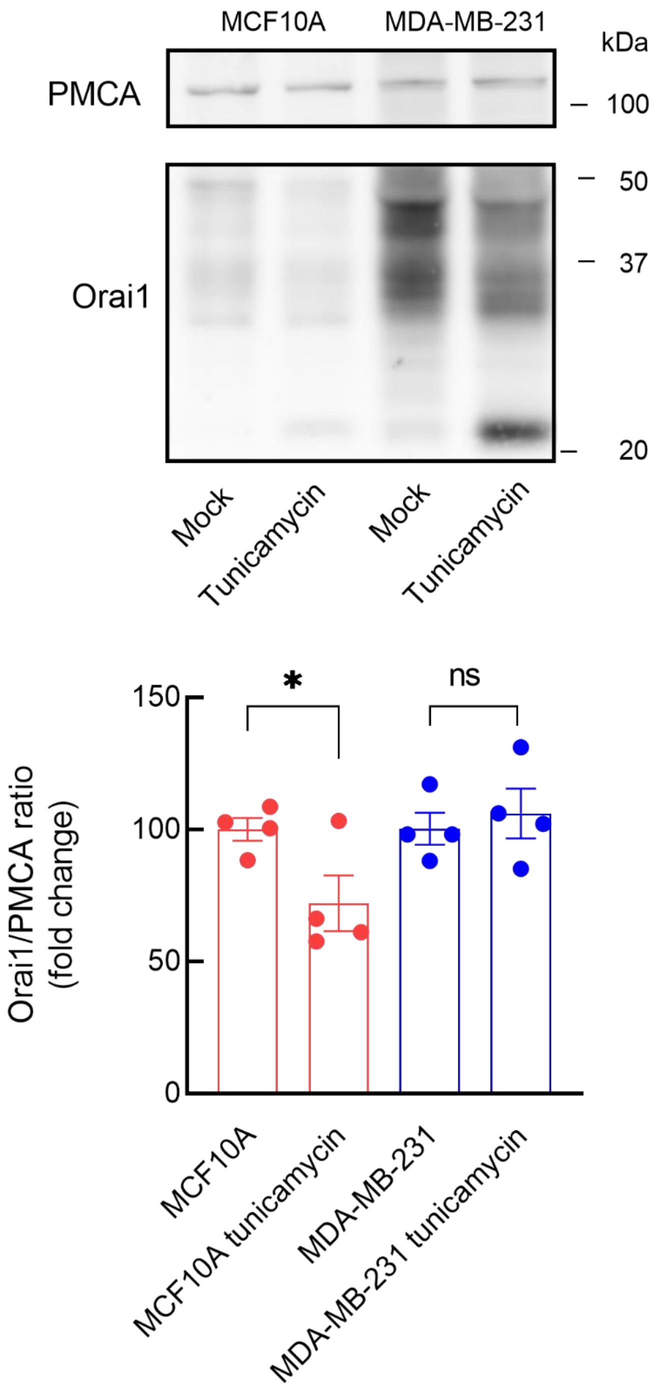

Figure S1. Effect of tunicamycin on Orai1 plasma membrane expression. MCF10A and MDA-MB-231 cells were incubated with 2 µM tunicamycin overnight or the vehicle (Mock), as indicated. Cells were mixed with biotinylation buffer containing EZ-Link sulfo-NHS-LC-biotin, and cell surface proteins were labeled by biotinylation, as described in "Material and methods". Labeled proteins were pulled down with streptavidin-coated agarose beads. The pellet (containing the plasma membrane fraction) was analyzed by SDS-PAGE and Western blotting using anti-Orai1 or anti-PMCA antibody, as indicated. Molecular masses indicated on the right were determined using molecular-mass markers run in the same gel. These results are representative of four separate experiments. Scatter plots represent the quantification of Orai1 plasma membrane expression under the different experimental conditions normalized to the PMCA expression and expresed as mean ± SEM. Data were statistically analyzed using Kruskal–Wallis test with multiple comparisons (Dunn's test). *$p < 0.05$ as compared to mock-treated MCF10A cells.

Table S1. Tunicamycin attenuates Orai1 and STIM1 protein expression in MCF10A and MDA-MB-231 cells.

	**Orai1**			
	control	tunicamycin	CHX	CHX+tunicamycin
MCF10A	1.00 ± 0.14	0.75 ± 0.12*	0.59 ± 0.14**	0.58 ± 0.14**
MDA-MB-231	1.00 ± 0.14	0.96 ± 0.09	0.75 ± 0.03**	0.71 ± 0.04**
		**STIM1**		
	control	tunicamycin	CHX	CHX+tunicamycin
MCF10A	1.00 ± 0.25	0.55 ± 0.19**	0.66 ± 0.14*	0.56 ± 0.18**
MDA-MB-231	1.00 ± 0.14	0.90 ± 0.21	0.91 ± 0.18	0.84 ± 0.16

MCF10A and MDA-MB-231 cells were incubated with 2 μM tunicamycin overnight or the vehicle (Mock) and further treated for 6h with 100 μg/mL cycloheximide (CHX) or the vehicle, as indicated, and lysed. Cell lysates were analyzed by Western blotting using specific anti-Orai1 antibody or anti-STIM1 antibody. The membrane was reprobed with anti-β-actin antibody for protein loading control. Data represents Orai1 and STIM1 expression normalized with β-actin and presented as fold-increase (experimental/control). Data are mean ± SEM of 4 independent experiments. Data were statistically analyzed using Kruskal–Wallis test with multiple comparisons (Dunn's test). * $p < 0.05$, ** $p < 0.01$ compared to control.
